# Effects of using pomegranate peel, beet leaf, and broccoli flower extracts on oxidative stability and physicochemical properties of cooked ground beef during refrigerated storage

**DOI:** 10.1002/fsn3.4419

**Published:** 2024-08-20

**Authors:** Sevgi Şimşek, Birol Kılıç

**Affiliations:** ^1^ Republic of Türkiye Ministry of Agriculture and Forestry, Food and Feed Division Isparta Turkey; ^2^ Faculty of Engineering, Department of Food Engineering Suleyman Demirel University Isparta Turkey

**Keywords:** antioxidant, ground beef, lipid oxidation, plant extracts

## Abstract

Plant extracts gained attention in the meat industry for their role in preventing microbial growth and oxidative deterioration. Antioxidant efficiency of various ratios (0.125, 0.25, 0.5, and 1%) of ethanolic or methanolic pomegranate peel (PP), beet leaf (BL), and broccoli flower (BF) extracts on cooked ground beef was investigated during refrigerated storage. Thiobarbituric acid reactive substances (TBARS), lipid hydroperoxide (LPO), p‐anisidine, cooking loss (CL), pH, and color analysis were performed. Results revealed that CL increased (*p* < .05) with 0.5 and 1% PE or 1% BF extracts, whereas no CL change was observed in other extract applications. Although using 0.5% and 1% of ethanolic or methanolic PP extracts caused lower pH than control (*p* < .05), no pH difference was found among other treatments. L* values were not affected by extract applications, whereas a* decreased and increased with PP and BL extract addition, respectively (*p* < .05). Meantime, a* values decreased and b* values increased by elevation in extract ratio (*p* < .05). In terms of solvent impact, methanolic PP and ethanolic BL extracts caused higher b* (*p* < .05), whereas no difference in b* was obtained between ethanolic or methanolic BL extracts. TBARS, LPO, and p‐anisidine analysis revealed that the highest lipid oxidation was obtained in control (*p* < .05). PP extract treatments exhibited the lowest lipid oxidation (*p* < .05). Lipid oxidation gradually increased in control and all extract‐incorporated samples during storage (*p* < .05). In extract‐incorporated samples, lipid oxidation decreased with increasing the extract ratio (*p* < .05). Lipid oxidation was inhibited more by all incorporation ratios for each tested extract compared to control (*p* < .05). Moreover, using 0.125% PP, 0.5% BL, or 1% BF extracts was more effective in lipid oxidation inhibition than BHT (*p* < .05). It may be concluded that PP, BL, and BF extracts may be utilized by the meat processors to achieve prolonged shelf life and improved quality features.

## Graphical abstract

It may be concluded that pomegranate peel, beet leaf, and broccoli flower extracts may be utilized by the meat processors to achieve prolonged shelf life and improved quality features.

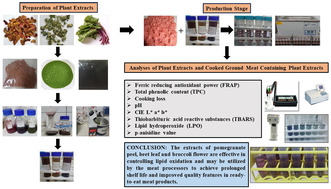

## 1 INTRODUCTION

Lipid oxidation is the most important chemical deterioration that causes quality losses, especially in thermally processed meat products (Kılıç et al., 2016). Lipid oxidation has negative effects on the quality of muscle foods by causing losses in sensory properties such as color, texture, taste, and smell, as well as in nutritional value, which may affect consumers' preferences (Domínguez et al., 2019). In addition, the degradation of fats in muscle foods also leads to the formation of toxic compounds which are stated to be associated with health problems such as atherosclerosis, Alzheimer, aging, and cancer (Lorenzo et al., 2018). In recent years, an increased awareness of the consumers for healthy eating habits has led the meat industry to focus on searching for alternative approaches in product formulations for the production of high‐quality and safe food with a long shelf life (Tenderis et al., 2020, 2021).

One of the most common methods to prevent or delay lipid oxidation is the addition of antioxidants (Şen & Kılıç, 2021). For this purpose, synthetic antioxidants such as butylated hydroxytoluene (BHT) and butylated hydroxyanisole (BHA) have been used for many years.

Although the use of synthetic antioxidants in industrial production is common practice, research on the use of natural antioxidants in muscle food formulations has increased due to reported toxic and carcinogenic effects of synthetic antioxidants and consumer demand for the use of natural additives in muscle food processing (Domínguez et al., 2019; Gόmez et al., 2018). Thus, in recent years, studies on the possible usage of spices, aromatic plants, fruit peels, and pulps and their extracts as natural antioxidant sources have gained importance (Uşan et al., 2022; Yuca et al., 2019). However, it is critical to know some issues when incorporation of plant extracts to have enhanced lipid oxidation inhibition in the meat products. Some factors seem to influence the antioxidant ability of plant extracts such as type of solvent used, extraction time, and temperature. Thus, optimum extraction conditions such as extraction temperature and time were assigned previously (Unpublished study) to obtain the highest antioxidant capacity in the tested plant extracts. Moreover, it is important to test the potential implementation of the plant extracts prepared by using previously determined optimized extraction conditions in the muscle foods to display enhanced lipid oxidation inhibition to create scientific infrastructure for industrial applications.

This research aimed to evaluate the antioxidant efficiency of various incorporation ratios of ethanolic or methanolic pomegranate peel, beet leaf, and broccoli flower extracts obtained by application of previously determined optimized extraction conditions for the highest antioxidant activity on cooked ground beef during 7‐day refrigerated storage.

## 2 MATERIALS AND METHODS

### 2.1 Materials

A 24‐h postmortem beef (*M. Longissimus thoracis et lumborum*) was obtained from a local slaughterhouse operating in Isparta province. After transferring to the laboratory under cold chain, the meat was ground, vacuum packaged and then stored at −20°C until used. Pomegranate (*Punica granatum*) peel, beet leaf, and broccoli (*Brassica oleracea* var. italica) flower were purchased from fruit and vegetable suppliers located in Isparta. In the analyses carried out within the scope of the study, Folin–Ciocalteu phenol reagent, gallic acid, sodium carbonate anhydrate, sodium acetate trihydrate, iron chloride hexahydrate, 2,4,6‐Tris (2‐pyridyl)‐s‐triazine, 37% hydrochloric acid, trichloroacetic acid, 2,2,4‐trimethylpentane, ethylene diamine tetraacetic acid, p‐anisidine, chloroform, sodium chloride, ethanol, cumene hydroperoxide, ammonium thiocyanate, iron(II) sulfate heptahydrate (Merck, Darmstadt, Germany), methanol (J.T. Baker, Deventer, Netherlands), 1,1‐diphenyl‐2‐picrylhydrazyl, thiobarbituric acid (Sigma‐Aldrich, Baden‐Württemberg, Germany), and propyl gallate (Thermo Fisher Scientific, New Jersey, United States) were used.

### 2.2 Preparation of plant extracts

The extraction process for each tested plant powder was carried out using the previously determined optimum conditions (Unpublished study) and the obtained extracts were stored at −80°C until used in ground meat. Twenty grams of each tested dried plant powder was taken and macerated with 100 mL of ethanol (v/v) or methanol (v/v) prepared at previously determined optimum concentrations for previously determined optimized extraction time and temperatures in a dark environment. At the extraction stage, applied extraction temperature, solvent concentration, and extraction time as optimum extraction conditions were 20°C, 40%, and 180 min for ethanolic extraction, 70°C, 76.36%, and 180 min for methanolic extraction of the pomegranate peel. These optimum extraction conditions were 70°C, 69.09%, and 180 min for ethanolic and 20°C, 64.24%, and 180 min for methanolic extraction of beet leaf. In broccoli flower, the optimum extraction conditions were 70°C, 73.33%, and 180 min for ethanolic and 20°C, 65.45%, and 180 min for methanolic extraction. At the end of the period, the mixture was filtered using Whatman No: 4 filter paper. The solvent was removed from the filtrate under vacuum in a rotary evaporator (Hei‐Vap, Heidolph, Germany) at 60°C until all of the alcohol was removed (Selani et al., 2011).

#### 2.2.1 Ferric reducing antioxidant power (FRAP) analysis

FRAP values of plant extracts were determined as described by Soyuçok et al. (2024). In brief, the FRAP reagent was made by mixing 300 mM acetate buffer with pH 3.6, 10 mM 2,4,6‐tripyridyl‐s‐triazine (Merck, Darmstadt, Germany) in 40 mmol HCl (Merck, Darmstadt, Germany), and 20 mmol ferric chloride (Merck, Darmstadt, Germany; 10:1:1). One hundred‐microliter tested extract and 3‐mL FRAP reagent were mixed up and the absorbance was measured by T8+ UV/VIS spectrometer (PG Instruments Ltd. England) at 593 nm. The results were expressed as mmol Fe^2+^ equivalents/g.

#### 2.2.2 Total phenolic content (TPC) analysis

The extract (1 mL) and 5 mL 0.2 N Folin–Ciocalteu reagent (Merck, Darmstadt, Germany) were mixed for 3 min. A 7.5% sodium carbonate (Merck, Darmstadt, Germany) was incorporated into the mixture and then kept for 30 min at room temperature. Absorbance was taken at 720 nm and the results were expressed as milligrams of gallic acid equivalents/gram (Goli et al., 2005).

### 2.3 Meat sample preparation and design of experimental groups

After removing from the deep freezer, ground beef was thawed at 4°C for 12 h. After incorporating 10% pure water and 2% NaCl, kneading was applied for 5 min. The kneaded ground meat was randomly divided into 26 treatments in equal portions and tested formulations were applied as presented in Table 1. The study included one control (without any tested plant extract or BHT) and one another treatment formulated with only BHT. Other 24 treatments were formed by incorporation of various incorporation ratios (0.125, 0.25, 0.5, and 1%) of ethanolic or methanolic PP, BL, and BF extracts. After carefully filling the ground meat (approximately 50 g) into plastic centrifuge tubes to have no air gap, the tubes were closed with screw caps and placed in a water bath set at 60°C and the temperature of the water bath was set at 85°C. The monitoring of the core temperature was carried out with a thermocouple. The thermal process was ended when the core temperature reached 74°C. After the thermal process, the cookout liquid was removed, and then cooked ground beef samples were weighed and stored at 4°C for 7 days. Color, pH, TBARS, LPO, and p‐anisidine analyses were performed on the samples for certain time intervals during storage.

**TABLE 1:** The treatments applied for formulation of ground beef experiments.

Treatments	
Control	No extract incorporation
BHT	0.01% BHT
EPP0.125	0.125% ethanolic pomegranate peel extract
EPP0.25	0.25% ethanolic pomegranate peel extract
EPP0.5	0.5% ethanolic pomegranate peel extract
EPP1	1% ethanolic pomegranate peel extract
MPP0.125	0.125% methanolic pomegranate peel extract
MPP0.25	0.25% methanolic pomegranate peel extract
MPP0.5	0.5% methanolic pomegranate peel extract
MPP1	1% methanolic pomegranate peel extract
EBL0.125	0.125% ethanolic beet leaf extract
EBL0.25	0.25% ethanolic beet leaf extract
EBL0.5	0.5% ethanolic beet leaf extract
EBL1	1% ethanolic beet leaf extract
MBL0.125	0.125% methanolic beet leaf extract
MBL0.25	0.25% methanolic beet leaf extract
MBL0.5	0.5% methanolic beet leaf extract
MBL1	1% methanolic beet leaf extract
EBF0.125	0.125% ethanolic broccoli flower extract
EBF0.25	0.25% ethanolic broccoli flower extract
EBF0.5	0.5% ethanolic broccoli flower extract
EBF1	1% ethanolic broccoli flower extract
MBF0.125	0.125% methanolic broccoli flower extract
MBF0.25	0.25% methanolic broccoli flower extract
MBF0.5	0.5% methanolic broccoli flower extract
MBF1	1% methanolic broccoli flower extract

#### 2.3.1 Cooking loss analysis

The raw ground meat weight (RMW) used for each treatment was recorded before processing. After the thermal process, the liquid part was removed from the tubes which were cooled at room temperature and the cooked ground meat sample weights (CMW) were recorded again. Cooking loss is calculated according to the formula shown below (Barbanti & Pasquini, 2005).

$$\text{Cooking loss}\;\left(\%\right)=\frac{\mathrm{RMW}-\mathrm{CMW}}{\mathrm{RMW}}\times 100$$

#### 2.3.2 pH and CIE L*a*b* color measurements

pH and CIE L*a*b* color measurements of the samples were carried out in triplicate for each treatment. For pH measurement, cooked ground meat samples (5 g) were homogenized in 50‐mL distilled water for 1 min. pH measurement was performed using a pH meter (HI 2211 pH/ORP meter, Hanna Instruments, Germany; AOAC, 1990). Calibration of the pH device was performed using pH 4.0–7.0 buffer solutions. CIE L* a* b* values were determined using a Minolta colorimeter (CR‐200, Illuminant D65, Minolta Corp., Ramsey, NJ, USA; Kılıç & Özer, 2019). Before the measurements, the device was calibrated using its own standard.

#### 2.3.3 Thiobarbituric acid reactive substances (TBARS) and lipid hydroperoxide (LPO) analysis

TBARS analysis was carried out as described by Özer and Kılıç (2020). EDTA and propyl gallate were added to the trichloroacetic acid (TCA) extraction solution to prevent the formation of TBARS during the analysis. Cooked ground meat sample (2 g) was mixed in 12 mL of extraction solution (7.5% TCA) and the samples were homogenized at 13,500 rpm for 15 s. The obtained homogenate was filtered through Whatman no: 1 filter paper. One milliliter of the obtained filtrate was mixed with thiobarbituric acid (TBA) solution and then vortexed. The resulting mixture was then heated in a block heater (Thermo Bath ALB FINEPCR, South Korea) at 100°C for 40 min. Then, the tubes were cooled in cold water. The cooled tubes were centrifuged at 4100 rpm for 10 min. Absorbance values were read at 532 nm against a blank which was prepared with 1 mL of TCA extraction solution and 1 mL of TBA solution. A standard curve was prepared using tetraethoxypropane (Merck, Germany) and analysis results were presented as μmol MDA/kg.

For LPO analysis, the method described by Kılıç et al. (2016) was used. In this method, 0.5 g of cooked ground meat sample was homogenized in 5 mL of chloroform/methanol (1:1) for 15 s. After adding 3.08 mL of 0.5% NaCl, the mixture was vortexed for 30 s. Then, this mixture was centrifuged at 2000 g for 6 min to achieve to have separation into two phases. Two milliliters of the lower phase was taken and added to the cold chloroform/methanol (1.3 mL; 1:1) mixture and vortexed. Twenty‐five microliters of ammonium thiocyanate (4.38 M) and 25 μL of iron (II) chloride (18 mM) were added and vortexed again for 4 s. After the samples were incubated for 20 min at room temperature, absorbance values were determined at 500 nm. For the determination of LPO values, a calibration curve was prepared using Cumene hydroperoxide (Merck, Darmstadt, Germany) and the results were presented as μmol/kg LPO.

#### 2.3.4 p‐anisidine value analysis

The p‐anisidine values were carried out to determine the amount of aldehydes, which are secondary oxidative degradation products. Analysis of p‐anisidine value in cooked ground meat samples was performed according to IUPAC (1987). The extraction of fats from cooked ground meat samples was carried out using the method explained by Bligh and Dyer (1959). 0.5 g of fat was dissolved in 25‐mL hexane and its absorbance (A1) was read by using a ultraviolet–visible spectrophotometer at 350 nm against hexane as a blank. This solution (5 mL) was mixed with 1 mL of 0.25% p‐anisidine solution for 10 min, and then the absorbance (A2) was recorded at 350 nm against hexane containing p‐anisidine. The p‐anisidine value was calculated using the equation shown below.

$$p-\text{anisidine value}=25\times \left[1.2\times \left(A2-\text{A1}\right)\right]/\left(\mathrm{fat}\;\text{weight}\right)$$

### 2.4 Statistical analysis

The entire study was replicated three times and the analyses performed in each replication were also carried out in triplicate. Differences among treatment groups were determined by using the SPSS 18 package program and using the analysis of variance (one‐way ANOVA). After applying the analysis of variance to the postproduction analyses (cooking loss, pH, instrumental color, TBARS, LPO, and p‐anisidine value), the differences among the treatment groups were determined by Duncan's multiple range test.

## 3 RESULTS AND DISCUSSION

### 3.1 Antioxidant capacity assay results of pomegranate peel, beet leaf, and broccoli flower extracts

TPC results of ethanolic or methanolic PP extracts at previously determined optimum conditions (20°C extraction temperature, 40% solvent concentration, and 180‐min extraction time for ethanolic extract; 70°C extraction temperature, 76.36% solvent concentration, and 180‐min extraction time for methanolic extract) were 123.72 ± 1.89 and 172.86 ± 1.09 mg GAE/g extract, respectively. On the other hand, FRAP results of ethanolic or methanolic PP extracts at previously determined optimum conditions were 1825.61 ± 1.87 and 1847.38 ± 0.88 mM Fe^+2^/g extract, respectively. Regarding BL extract, TPC values of ethanolic or methanolic extracts at previously determined optimum conditions (70°C extraction temperature, 69.09% solvent concentration, and 180‐min extraction time for ethanolic extract; 20°C extraction temperature, 64.29% solvent concentration, and 180‐min extraction time for methanolic extract) were 48.77 ± 2.79 and 57.73 ± 1.28 mg GAE/g extract, respectively. FRAP results of ethanolic or methanolic BL extracts at previously determined optimum conditions were 601.57 ± 1.45 and 609.26 ± 0.79 mM Fe^+2^/g extract, respectively. As far as BF extract is concerned, TPC values of ethanolic or methanolic extracts at previously determined optimum conditions (70°C extraction temperature, 73.33% solvent concentration, and 180‐min extraction time for ethanolic extract; 20°C extraction temperature, 65.45% solvent concentration, and 180‐min extraction time for methanolic extract) were 51.94 ± 1.37 and 38.55 ± 1.64 mg GAE/g extract, respectively. FRAP results of ethanolic or methanolic BF extracts at previously determined optimum conditions were 617.63 ± 0.71 and 424.18 ± 0.79 mM Fe^+2^/g extract, respectively. Similar results regarding TPC obtained from PP extracts were reported by Djenidi et al. (2020) and Fawole et al. (2012). On the other hand, Derakhshan et al. (2018) showed that TPC values obtained from PP extracts were higher than the values obtained in our study. It is thought that this difference in results may be due to the difference in raw materials and extraction conditions. Fernández et al. (2017) found that TPC obtained from BL was 305.8 mg/kg and reported that TPC obtained from BL was higher than that of many vegetables. Deng et al. (2013) stated that BF contains high amounts of TPC. A similar findings regarding FRAP values obtained from PP were also reported by Guo et al. (2003). Researchers stated that the highest FRAP value (82.11 mmol/100 g fresh weight) among 28 tested fruits was obtained in PP. Wang et al. (2011) stated that PP contains high amounts of phenolic compounds such as tannins and anthocyanins and therefore has high antioxidant activity. Elshiemy et al. (2018) also reported that the extract obtained from BL had high FRAP values. Djenidi et al. (2020) found IC50 value as 0.32 mg/mL in pomegranate extract and 1.76 mg/mL in beetroot extract.

### 3.2 Effects of pomegranate peel, beet leaf, and broccoli flower extracts on lipid oxidation inhibition and physicochemical features of cooked ground beef during refrigerated storage

#### 3.2.1 Cooking loss results

**FIGURE 1:**
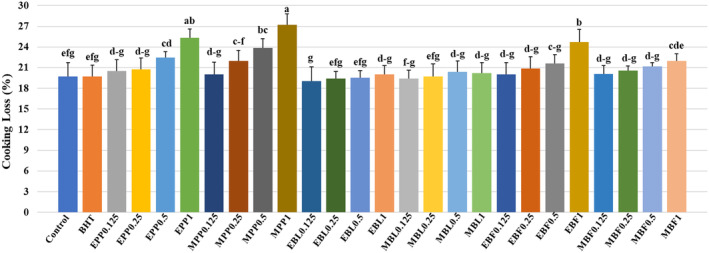
Influences of plant extracts on cooking loss values of cooked ground beef.

CL values of 26 treatments (Figure 1) were found to be ranged from 19.05 to 27.20%. It was observed that CL values of the treatments containing 0.5% or 1% ethanolic or methanolic PP extract and 1% ethanolic BF extract were higher compared to those of control and BHT treatment (*p* < .05). Our results were supported by Hama et al. (2018) who has also been reported an increased CL values with the addition of PP extract. Researchers emphasized that a decrease in pH due to the addition of PP extract may cause an increase in CL. On the other hand, CL values determined in the other treatments are similar to the values determined in control and BHT treatment. Banerjee et al. (2012) also reported that there was no significant difference in CL between the samples containing broccoli powder extract and control. In addition, our results demonstrated that even though the incorporation of ethanolic PP or BF extracts up to 0.5% did not create a significant change in CL, CL values increased with the addition of 1% ethanolic PP or BF extracts (*p* < .05). On the other hand, our results revealed that a gradual increase in CL was obtained with increasing extract incorporation dose in the treatments containing methanolic PP extract (*p* < .05). However, this relationship between extract incorporation ratio and CL was not exist in the case of ethanolic or methanolic BL and methanolic BF extracts which means that the extract incorporation ratio was not a factor affecting CL in cooked ground beef for these treatments.

#### 3.2.2 pH and CIE L*a*b* color measurement results

pH and CIE L*a*b* color measurement results are presented in Table 2. pH values of 26 treatments varied between 5.69 and 5.87 on processing day. Furthermore, EPP1, MPP0.5, and MPP1 treatments had lower (*p* < .05) pH values than those determined in control on processing day, whereas there were no pH differences between the other treatments and control. Similarly, Hama et al. (2018) stated that the incorporation of PP extract into lamb caused a decrease in pH. Banerjee et al. (2012) reported that there was no significant influence on pH when nuggets were manufactured with broccoli extract incorporation up to 1%; however, authors reported a pH decline in nuggets at 1.5 and 2% extract incorporation levels. In addition, Lee and Chin (2012) stated that utilization of red beet extract in pork meatball processing did not create a significant change in pH. In our study, pH increase was observed in all treatments after 1 day of storage (*p* < .05). Ibrahim et al. (2010) also observed an increasing trend in pH during refrigerated storage in lamb patties and researchers stated that an increase in pH during the storage was associated with ammonia accumulation as a result of protein denaturation. Elevation of pH during refrigerated storage was also reported by other previous studies in pork meatballs (Lee & Chin, 2012), lamb (Hama et al., 2018), and PP extract‐incorporated chicken meatballs (Sharma & Yadav, 2020). On the first day of storage, EPP0.5, EPP1, MPP0.5, MPP1, MBF0.5, and MBF1 were found to have lower (*p* < .05) pH values compared to control and BHT treatments which were quite similar with the rest of the treatments. No significant changes were observed in pH values during the rest of the storage period for all treatments except MBF0.5 and MBF1 treatments where pH gradually increased during the same period of time (*p* < .05). At the end of storage, pH values obtained in EPP0.5, EPP1, MPP0.5, and MPP1 treatments were lower (*p* < .05) than those obtained in control and BHT treatments, and there was no pH difference between these two treatments and the rest of the other treatments regarding pH. In general, results revealed nonsignificant pH changes in cooked ground meat associated with an increase in the incorporation ratio of all tested plant extracts.

**TABLE 2:** Effects of plant extracts on pH in cooked ground beef during 7‐day storage at 4°C.

Treatments	Processing day	1 day	7 days
Control	5.87 ± 0.06^aB^	6.00 ± 0.01^a–dA^	6.04 ± 0.03^abA^
BHT	5.84 ± 0.07^abB^	6.00 ± 0.03^abcA^	6.03 ± 0.05^abA^
EPP0.125	5.80 ± 0.09^abcB^	5.99 ± 0.03^a–dA^	6.00 ± 0.04^a–dA^
EPP0.25	5.79 ± 0.08^a–dB^	5.95 ± 0.02^defA^	5.98 ± 0.06^a–eA^
EPP0.5	5.76 ± 0.07^a–dB^	5.91 ± 0.05^fgA^	5.92 ± 0.05^defA^
EPP1	5.71 ± 0.08^cdB^	5.89 ± 0.06^gA^	5.90 ± 0.07^fA^
MPP0.125	5.82 ± 0.08^abcB^	5.96 ± 0.02^b–eA^	5.97 ± 0.06^b–eA^
MPP0.25	5.78 ± 0.05^a–dB^	5.95 ± 0.02^defA^	5.98 ± 0.05^b–eA^
MPP0.5	5.74 ± 0.08^bcdB^	5.94 ± 0.03^efA^	5.94 ± 0.04^c–fA^
MPP1	5.69 ± 0.07^dB^	5.91 ± 0.02^fgA^	5.91 ± 0.02^efA^
EBL0.125	5.82 ± 0.09^abB^	6.01 ± 0.00^abA^	6.02 ± 0.02^abA^
EBL0.25	5.84 ± 0.08^abB^	6.00 ± 0.01^abcA^	6.02 ± 0.04^abA^
EBL0.5	5.84 ± 0.06^abB^	6.01 ± 0.03^abA^	6.02 ± 0.05^abcA^
EBL1	5.85 ± 0.08^abB^	6.04 ± 0.02^aA^	6.06 ± 0.04^aA^
MBL0.125	5.83 ± 0.07^abB^	6.01 ± 0.05^abA^	6.02 ± 0.07^abA^
MBL0.25	5.81 ± 0.09^abcB^	6.00 ± 0.03^abcA^	6.02 ± 0.07^abcA^
MBL0.5	5.82 ± 0.09^abcB^	6.01 ± 0.03^abA^	6.01 ± 0.07^abcA^
MBL1	5.83 ± 0.08^abB^	6.00 ± 0.04^a–dA^	6.01 ± 0.05^abcA^
EBF0.125	5.82 ± 0.07^abcB^	5.97 ± 0.04^b–eA^	5.98 ± 0.07^a–eA^
EBF0.25	5.81 ± 0.07^abcB^	5.97 ± 0.04^b–eA^	6.00 ± 0.06^a–dA^
EBF0.5	5.82 ± 0.10^abcB^	5.96 ± 0.04^b–eA^	5.99 ± 0.07^a–dA^
EBF1	5.80 ± 0.11^abcB^	5.95 ± 0.05^c–fA^	5.99 ± 0.07^a–eA^
MBF0.125	5.81 ± 0.09^abcB^	5.96 ± 0.04^b–eA^	5.99 ± 0.06^a–eA^
MBF0.25	5.82 ± 0.09^abcB^	5.96 ± 0.04^b–eA^	5.98 ± 0.06^a–eA^
MBF0.5	5.82 ± 0.06^abcC^	5.93 ± 0.06^efgB^	6.00 ± 0.05^abcA^
MBF1	5.82 ± 0.07^abcC^	5.94 ± 0.04^efB^	6.00 ± 0.07^abcA^

*Note*: Mean ± SD; ^a–g^Within a column, values superscripted with different letters are significantly different (*p* < .05). ^A–C^Within a row, values superscripted with different letters are significantly different (*p* < .05).

Color measurement results (Table 3) showed that L* values ranged between 50.72 and 54.69 on processing day. Results revealed that plant extract incorporation ratio was not a factor creating significant changes in L* values on processing day and each stage of storage for all tested plant extracts. Although lower L* values were determined in EBL1 compared to control on processing day, EBL1 and EBF1 treatments had lower L* values compared to BHT treatment (*p* < .05). Similarly, the usage of PP extract was also reported to decrease L* values in Weiners (Zago et al., 2020) and cooked goat meat patties (Devatkal et al., 2010). Marrone et al. (2021) also stated a decrease in L* values of beef burgers as a result of beet extract application. There was no significant change in L* values of all treatments during storage. When the changes in L* values on the first day of storage were examined, L* values of all treatments containing 1% extract were found to be lower than L* values of control and BHT treatments (*p* < .05). At the end of the storage, although L* values of EBL1 and MBL1 treatments were lower than those of BHT treatment (*p* < .05), generally no significant difference was found among the other treatments. It was determined that a* values changed between 9.48 and 17.19 on processing day and 8.06 and 15.67 at the end of storage. The lowest a* values were determined in treatments containing 1% methanolic PP extract or 1% ethanolic BF extract on processing day, while the highest a* values were determined in treatments containing 0.5% and 1% methanolic BL extract (*p* < .05). Zago et al. (2020) reported a decrease in a* values of Weiners due to the incorporation of PP extract and authors emphasized that a decrease in a* values was enhanced with increasing the incorporation ratio of the extract. Similarly, it was stated that beef burgers containing PP extract had lower a* values (Shahamirian et al., 2019). Marrone et al. (2021), on the other hand, stated that red beet extract caused an increase in a* values of beef burgers and a* values increased as the amount of added extract increased. On the other days of storage, the lowest a* value was determined in treatments containing 1% ethanolic BF extract (*p* < .05). While the highest a* values were determined in the MBL1 on the first day of storage, the highest a* value was determined in MBL0.125 at the end of storage (*p* < .05). In general, there was a significant decrease in a* values at the end of storage in all treatments except BHT, EPP1, MPP1, and MBL0.125 (*p* < .05). Shahamirian et al. (2019) reported a gradual decrease in a* values during storage in beef burgers and this was explained with development of metmyoglobin formation. Results indicated that a* values obtained in BHT, EPP1, MPP1, and MBL0.125 treatments did not show a significant change during storage. In general, lower a* values were obtained in treatments containing PP extract compared to control during the storage period (*p* < .05). Higher a* values were determined in the treatments containing the methanolic BL extracts compared to control (*p* < .05). Even though the use of ethanolic BL extracts at all incorporation ratios resulted in higher a* values compared to control on processing day, cooked ground beef formulated with 0.125% and 0.25% incorporation ratios had a higher and those formulated with 0.5% and 1% had lower a* values than control during the storage period (*p* < .05). As a result, using 0.5% and 1% of BF extract created lower a* values compared to control (*p* < .05). Similarly, Kumar et al. (2013) reported that lower a* values were obtained in nuggets containing broccoli powder.

**TABLE 3:** Effects of plant extract on CIE L*a*b* color values in cooked ground beef during 7‐day storage at 4°C.

Treatments	L*			a*			b*		
	Processing day	1 day	7 days	Processing day	1 day	7 days	Processing day	1 day	7 days
Control	54.17 ± 2.42^abA^	56.34 ± 3.39^abA^	54.16 ± 3.08^a–dA^	13.97 ± 0.87^fghA^	13.71 ± 0.38^fgA^	12.69 ± 0.31^deB^	3.32 ± 0.24^k–nB^	2.90 ± 0.32^noB^	3.88 ± 0.42^hiA^
BHT	54.69 ± 2.38^aB^	57.57 ± 2.13^aA^	56.77 ± 2.90^aAB^	14.30 ± 0.82^efgA^	13.96 ± 0.25^efgA^	13.74 ± 0.32^cA^	3.87 ± 0.21^g–mA^	3.62 ± 0.35^jklAB^	3.32 ± 0.10^ijkB^
EPP0.125	53.38 ± 1.35^abA^	55.66 ± 3.17^abcA^	54.44 ± 1.79^a–dA^	13.75 ± 0.64^ghA^	13.54 ± 0.94^fghA^	11.73 ± 0.71^ghiB^	3.99 ± 0.40^g–kB^	3.94 ± 0.21^ijB^	4.53 ± 0.35^gA^
EPP0.25	52.62 ± 4.75^abcA^	52.57 ± 0.81^efgA^	56.62 ± 3.60^a–dA^	13.53 ± 0.67^hiA^	13.05 ± 0.77^hiA^	11.60 ± 0.76^ghiB^	2.62 ± 0.18°^C^	3.84 ± 0.24^ijkA^	3.33 ± 0.12^ijkB^
EPP0.5	54.62 ± 1.16^aA^	55.40 ± 1.52^a–dA^	54.12 ± 1.83^a–dA^	12.84 ± 0.75^ijA^	11.96 ± 0.50^klB^	11.77 ± 0.54^ghiB^	3.92 ± 0.38^g–lB^	4.67 ± 0.26^fgA^	4.44 ± 0.36^gAB^
EPP1	54.22 ± 2.35^abA^	54.63 ± 1.54^b–eA^	56.04 ± 2.63^abA^	11.51 ± 0.54^klA^	11.54 ± 0.47^lmA^	11.19 ± 0.29^ijA^	5.84 ± 0.38^cdA^	5.34 ± 0.20^eA^	5.47 ± 0.29^fAB^
MPP0.125	53.85 ± 2.17^abA^	54.02 ± 2.54^b–fA^	54.21 ± 3.67^a–dA^	13.58 ± 0.67^ghiA^	12.71 ± 0.39^ijB^	12.01 ± 0.42^fgC^	3.21 ± 0.39^mnoB^	3.99 ± 0.41^hijA^	3.86 ± 0.23^hiA^
MPP0.25	54.30 ± 1.74^abA^	55.33 ± 1.59^a–dA^	54.46 ± 1.94^a–dA^	12.99 ± 0.48^iA^	12.02 ± 0.69^klB^	11.39 ± 0.34^hijC^	3.45 ± 0.36^k–nA^	3.49 ± 0.44^j–nA^	3.51 ± 0.39^ijA^
MPP0.5	53.38 ± 2.65^abA^	55.63 ± 1.09^c–fA^	55.17 ± 1.95^abcA^	11.37 ± 0.64^lA^	11.18 ± 0.56^mA^	10.61 ± 0.41^klB^	6.26 ± 0.77^bcAB^	5.45 ± 0.28^eB^	6.86 ± 0.32^dA^
MPP1	53.92 ± 3.40^abA^	55.80 ± 1.53^abcA^	54.31 ± 1.86^a–dA^	10.06 ± 0.37^mA^	10.25 ± 0.49^nA^	9.81 ± 0.37^mA^	6.63 ± 0.71^bB^	7.09 ± 0.59^cB^	9.48 ± 0.37^bA^
EBL0.125	53.24 ± 2.10^abcA^	55.03 ± 2.44^a–eA^	54.02 ± 3.28^a–dA^	14.92 ± 0.43^cdeA^	14.77 ± 0.59^dA^	13.66 ± 0.39^cB^	3.56 ± 0.14^j–nA^	2.96 ± 0.23^mnoB^	2.99 ± 0.24^jklB^
EBL0.25	54.36 ± 1.85^aA^	54.49 ± 3.02^b–fA^	53.23 ± 2.95^bcdA^	14.81 ± 0.85^cdeA^	14.42 ± 0.55^deB^	14.13 ± 0.29^cB^	3.43 ± 0.25^k–nA^	3.23 ± 0.37^l–oA^	3.39 ± 0.25^ijkA^
EBL0.5	52.44 ± 1.57^abcA^	52.92 ± 2.79^defA^	53.48 ± 2.18^bcdA^	14.82 ± 0.48^cdeA^	12.19 ± 0.63^jklB^	11.49 ± 0.64^ghiC^	4.44 ± 0.42^fghB^	5.38 ± 0.52^eA^	5.63 ± 0.15^efA^
EBL1	50.72 ± 1.97^cA^	50.31 ± 0.80^gA^	51.72 ± 1.95^dA^	15.08 ± 0.40^cdA^	12.04 ± 0.68^jklB^	11.94 ± 0.85^fghB^	5.80 ± 0.51^ceB^	6.11 ± 0.54^dAB^	6.83 ± 0.44^dA^
MBL0.125	54.38 ± 1.20^aA^	54.00 ± 1.49^b–fA^	54.59 ± 1.17^a–dA^	15.54 ± 0.67^cA^	15.51 ± 0.74^cA^	15.67 ± 0.44^aA^	3.05 ± 0.41^noA^	2.88 ± 0.26^oAB^	2.49 ± 0.23^lA^
MBL0.25	52.37 ± 2.29^abcA^	53.71 ± 1.84^b–fA^	54.29 ± 1.99^a–dA^	16.31 ± 0.78^bA^	16.20 ± 0.79^abA^	15.25 ± 0.71^abB^	3.75 ± 0.41^i–mA^	3.50 ± 0.31^j–nAB^	2.92 ± 0.37^klB^
MBL0.5	53.52 ± 0.73^abA^	52.79 ± 2.71^defA^	53.06 ± 2.87^bcdA^	16.94 ± 0.62^abA^	15.77 ± 0.60^bcB^	14.14 ± 0.39^cC^	4.52 ± 0.43^fgA^	4.41 ± 0.16^ghiA^	4.29 ± 0.25^ghA^
MBL1	52.32 ± 1.16^abcA^	51.91 ± 3.16^fgA^	52.70 ± 1.70^cdA^	17.19 ± 1.40^aA^	16.74 ± 0.81^aA^	14.81 ± 0.76^bB^	5.23 ± 0.30^deA^	5.50 ± 0.58^eA^	6.01 ± 0.59^eA^
EBF0.125	53.19 ± 2.45^abcB^	53.95 ± 2.53^bfAB^	56.61 ± 2.06^aA^	15.13 ± 0.65^cdA^	14.18 ± 0.77^defB^	13.76 ± 0.66^cB^	3.24 ± 0.39^l–oA^	3.51 ± 0.40^j–mA^	3.21 ± 0.08^jkA^
EBF0.25	53.49 ± 1.02^abA^	55.08 ± 2.36^a–eA^	53.87 ± 4.04^a–dA^	13.41 ± 0.83^hiA^	12.25 ± 0.65^jkB^	12.40 ± 0.37^efB^	4.99 ± 0.38^efA^	5.07 ± 0.46^efA^	5.25 ± 0.49^fA^
EBF0.5	54.58 ± 2.12^aA^	55.16 ± 0.95^a–eA^	54.43 ± 0.91^a–dA^	12.19 ± 0.33^jkA^	12.14 ± 0.44^jklA^	10.11 ± 0.60^lmB^	6.56 ± 0.52^bB^	8.17 ± 0.68^bA^	8.18 ± 0.74^cA^
EBF1	51.60 ± 2.14^bcB^	53.33 ± 1.03^c–fA^	53.94 ± 1.21^a–dA^	9.48 ± 0.57^mA^	8.89 ± 0.51^oB^	8.06 ± 0.47^nC^	8.58 ± 0.76^aB^	9.37 ± 0.40^aB^	10.57 ± 0.67^aA^
MBF0.125	53.50 ± 2.18^abA^	55.79 ± 2.78^abcA^	54.66 ± 3.80^a–dA^	14.59 ± 0.48^defA^	13.42 ± 0.53^ghB^	13.11 ± 0.45^dB^	3.80 ± 0.33^h–mA^	3.29 ± 0.13^k–oB^	2.99 ± 0.19^jklB^
MBF0.25	52.14 ± 2.74^abcB^	55.38 ± 1.30^a–dA^	55.22 ± 1.81^abcA^	13.94 ± 0.47^fghA^	13.04 ± 0.27^hiB^	12.68 ± 0.42^deB^	3.38 ± 0.23^f–iA^	3.86 ± 0.28^ijkB^	3.05 ± 0.21^jkC^
MBF0.5	52.77 ± 0.37^abcA^	54.28 ± 3.01^b–fA^	55.33 ± 3.64^abcA^	12.93 ± 0.35^iA^	11.51 ± 0.69^lmB^	10.86 ± 0.40^jkC^	4.15 ± 0.24^g–jB^	4.53 ± 0.26^fghA^	4.49 ± 0.06^gA^
MBF1	53.94 ± 2.54^abA^	53.51 ± 1.76^c–fA^	53.01 ± 1.39^bcdA^	11.61 ± 0.74^klA^	9.96 ± 0.55^nB^	9.93 ± 0.43^mB^	5.33 ± 0.29^deB^	6.04 ± 0.14^dA^	5.74 ± 0.22^efA^

*Note*: Mean ± SD; ^a–g^Within a column, values superscripted with different letters are significantly different (*p* < .05). ^A–C^Within a row, values superscripted with different letters are significantly different (*p* < .05).

While b* values varied between 2.62 and 8.58 on processing day, they varied between 2.49 and 10.57 at the end of storage. Generally, the highest b* values were found in the treatments containing 1% ethanolic BF extract on all storage days (*p* < .05). The lowest b* values were generally found in MBL0.125 (*p* < .05). In general, an increase in b* values was observed in all treatments as a result of an increase in the incorporation ratio of all tested plant extracts (*p* < .05). A similar trend was reported by Turgut et al. (2017) in meatball samples containing PP extract. In general, as a result of using ethanolic PP extract, higher b* values were obtained compared to control (*p* < .05). It was observed that methanolic PP and BL extracts and ethanolic BL extract used at incorporation ratios of 0.5% and 1% resulted in higher b* values than control (*p* < .05). Turgut et al. (2017) reported that the usage of 1% PP extract resulted in an increase in the yellowness of meatballs. Choi et al. (2017) stated that b* values of cooked meat emulsions containing 10% fermented beet extract were higher than control. On the other hand, Marrone et al. (2021) stated that lower yellowness values were obtained in burgers containing beetroot extract. In the treatments containing BF extract, using 0.25% or higher extract incorporation ratios resulted in higher b* values compared to control (*p* < .05).

#### 3.2.3 TBARS and LPO

The results of TBARS changes during the storage of cooked ground beef at 4°C are presented in Table 4. The highest TBARS values were obtained in control on both processing day and storage period (*p* < .05). On processing day, TBARS values of all treatments containing ethanolic or methanolic extracts of PP and BL (except MBL0.125) were lower than TBARS values obtained in BHT treatment (*p* < .05). TBARS values of treatments containing 0.5% or 1% ethanolic or methanolic extracts of BF were also found to be lower than the values obtained in BHT treatment (*p* < .05). Inhibition of TBARS formation due to PP extract was reported previously in cooked lamb (Hama et al., 2018) and goat meatballs (Devatkal et al., 2010). On the other hand, the reduction in TBARS formation was stated in goat meat nuggets containing broccoli powder extract (Banerjee et al., 2012) and beef burgers containing beetroot extract (Marrone et al., 2021).

**TABLE 4:** Effects of plant extracts on TBARS values in cooked ground beef during 7‐day storage at 4°C.

Treatments	Processing day	1 day	7 days
Control	5.08 ± 0.29^aC^	13.49 ± 0.81^aB^	31.25 ± 2.81^aA^
BHT	3.41 ± 0.21^cdC^	7.04 ± 0.57^eB^	18.24 ± 1.78^gA^
EPP0.125	2.06 ± 0.31^lmC^	4.04 ± 0.39^hB^	7.30 ± 0.83^kA^
EPP0.25	1.94 ± 0.16^mnoC^	2.62 ± 0.32^lB^	4.61 ± 0.45^lmA^
EPP0.5	1.70 ± 0.13^noB^	1.88 ± 0.14^mAB^	2.12 ± 0.37^opA^
EPP1	1.64 ± 0.19^noA^	1.60 ± 0.21^mA^	1.70 ± 0.11^opA^
MPP0.125	2.40 ± 0.24^jklC^	3.82 ± 0.45^hiB^	9.44 ± 0.39^iA^
MPP0.25	1.99 ± 0.30^mnB^	2.00 ± 0.47^mB^	2.82 ± 0.18^noA^
MPP0.5	1.60 ± 0.30^oA^	1.63 ± 0.12^mA^	1.50 ± 0.18^opA^
MPP1	1.67 ± 0.17^noA^	1.52 ± 0.27^mAB^	1.25 ± 0.22^pB^
EBL0.125	2.47 ± 0.11^ijkC^	7.52 ± 0.18^deB^	24.39 ± 1.05^dA^
EBL0.25	2.35 ± 0.26^jklC^	5.02 ± 0.44^gB^	20.09 ± 0.16^fA^
EBL0.5	2.39 ± 0.13^jklC^	3.04 ± 0.25^jklB^	12.25 ± 0.53^hA^
EBL1	2.94 ± 0.35^efgB^	2.83 ± 0.14^klB^	3.70 ± 0.42^mnA^
MBL0.125	3.17 ± 0.41^deC^	7.88 ± 0.36^dB^	26.11 ± 0.83^cA^
MBL0.25	2.62 ± 0.35^g–jC^	5.74 ± 0.33^fB^	20.13 ± 1.12^fA^
MBL0.5	2.56 ± 0.24^h–kB^	3.35 ± 0.39^ijB^	11.84 ± 0.85^hA^
MBL1	2.90 ± 0.21^e–hB^	2.86 ± 0.10^jklB^	5.29 ± 0.40^lA^
EBF0.125	3.90 ± 0.28^bC^	8.49 ± 0.44^cB^	27.33 ± 1.21^bcA^
EBF0.25	3.11 ± 0.26^defC^	7.44 ± 0.60^deB^	24.02 ± 1.12^deA^
EBF0.5	2.51 ± 0.27^ijkC^	4.89 ± 0.32^gB^	17.65 ± 0.92^gA^
EBF1	2.26 ± 0.05^klmC^	3.23 ± 0.26^jkB^	8.75 ± 0.42^ijA^
MBF0.125	3.66 ± 0.45^bcC^	10.84 ± 0.32^bB^	27.71 ± 1.58^bA^
MBF0.25	3.82 ± 0.16^bC^	7.61 ± 0.40^dB^	22.80 ± 0.92^eA^
MBF0.5	2.76 ± 0.14^f–iC^	6.03 ± 0.25^fB^	17.86 ± 0.87^gA^
MBF1	2.46 ± 0.039^ijkB^	2.98 ± 0.35^jklB^	7.75 ± 0.59^jkA^

*Note*: Mean ± SD; ^a–g^Within a column, values superscripted with different letters are significantly different (*p* < .05). ^A–C^Within a row, values superscripted with different letters are significantly different (*p* < .05).

On the first day of storage, TBARS values of all treatments except EBL0.125, MBL0.125, EBF0.125, EBF0.25, MBF0.125, and MBF0.25 were found to be lower than those determined in BHT treatment (*p* < .05). Lee and Chin (2012) noted that TBARS determined in pork patties containing beet extract was lower than control. TBARS values increased gradually during storage in all treatments except EPP1, MPP0.5, and MPP1 (*p* < .05). Similarly, an increase in TBARS during storage of the meat products formulated with plant extracts has also been reported (Jahan et al., 2018; Marrone et al., 2021). Regarding EPP1, MPP0.5, and MPP1 treatments, TBARS values were quite stable in EPP1 and MPP0.5 treatments during storage, whereas TBARS values gradually decreased in MPP1 at the same period of storage (*p* < .05). Hama et al. (2018) reported that even though TBARS in lamb containing 1% pomegranate extract did not change during storage, the addition of 1.5% pomegranate extract caused a gradual decrease in TBARS during storage. At the end of the storage, lower TBARS values were determined in all treatments containing PP extract, in the treatments containing 0.5% and 1% ethanolic or methanolic extracts of BL, and in the treatments containing 1% ethanolic or methanolic BF extract compared to BHT treatment (*p* < .05). Moreover, TBARS values obtained in EBF0.5 and MBF0.5 treatments were similar to TBARS determined in BHT treatment at the end of storage. In the samples containing ethanolic or methanolic extracts of PP extract, TBARS values decreased gradually with an increase in extract incorporation ratio up to 0.5% (*p* < .05). However, there was no difference between treatments containing 0.5% or 1% PP extract in terms of TBARS. On the other hand, a gradual decrease in TBARS was determined with an increase in extract incorporation ratio in the treatments containing BL or BF extracts (*p* < .05). Similarly, Zago et al. (2020) stated that TBARS values decreased with the increase in extract incorporation ratio in Weiners containing PP extract. When the plant extracts were compared among themselves, the lowest TBARS values were obtained in treatments containing PP extract, while the highest TBARS values were obtained in treatments containing BF extract (*p* < .05). As far as solvent type is concerned, in general, no significant differences were found in terms of TBARS values among the treatments containing plant extracts whether or not obtained by using ethyl or methyl alcohol for extraction.

The results of LPO changes during the storage of cooked ground beef meat at 4°C are presented in Table 5. LPO values on processing day ranged between 29.20 and 59.24 μmol/kg LPO, whereas LPO values were changed between 37.04 and 204.15 μmol/kg LPO at the end of the storage. The highest LPO values were determined in control on all storage days (*p* < .05). While the lowest LPO values were determined in treatment containing 1% methanolic PP extract on processing day, LPO values of the treatments containing 0.5% or 1% ethanolic or methanolic extracts of PP were found to be lower than LPO values determined in the other treatments (*p* < .05). During the whole storage period, LPO values of the treatments containing PP extract were lower than the values obtained in BHT treatments (*p* < .05). Similarly, Jahan et al. (2018) stated that the peroxide values of meatballs containing PP extract were lower than control and meatballs containing BHA. Shahamirian et al. (2019), on the other hand, noted that beef burgers containing PP extract had similar peroxide values with BHT‐containing samples and lower peroxide values than control and the samples containing pomegranate juice.

**TABLE 5:** Effects of plant extracts on LPO values in cooked ground beef during 7‐day storage at 4°C.

Treatments	Processing day	1 day	7 days
Control	59.24 ± 4.19^aC^	85.39 ± 3.89^aB^	204.15 ± 17.63^aA^
BHT	45.69 ± 3.30^f–iC^	58.73 ± 3.87^efB^	113.41 ± 10.19^ghA^
EPP0.125	40.63 ± 3.42^jkC^	48.36 ± 3.81^gB^	73.24 ± 4.91^kA^
EPP0.25	40.38 ± 3.21^jkB^	44.63 ± 1.15^ghB^	61.70 ± 4.36^lA^
EPP0.5	33.57 ± 1.60^lB^	34.89 ± 1.25^jkB^	40.96 ± 3.38^nA^
EPP1	36.58 ± 1.97^klA^	34.14 ± 2.02^jkA^	38.25 ± 3.32^nA^
MPP0.125	40.40 ± 1.84^jkC^	49.09 ± 5.23^gB^	85.90 ± 6.98^ijA^
MPP0.25	40.26 ± 3.02^jkB^	43.28 ± 2.10^hiB^	52.01 ± 2.91^lmA^
MPP0.5	36.59 ± 1.30^klB^	39.05 ± 3.40^ijAB^	43.16 ± 3.97^mnA^
MPP1	29.20 ± 2.97^mB^	31.06 ± 2.44^kB^	37.04 ± 3.66^nA^
EBL0.125	45.21 ± 3.51^ghiC^	58.49 ± 1.49^efB^	136.92 ± 6.90^deA^
EBL0.25	43.00 ± 2.58^ijC^	56.24 ± 3.98^fB^	114.07 ± 6.32^ghA^
EBL0.5	43.05 ± 2.73^g–jC^	55.63 ± 4.22^fB^	94.74 ± 8.28^iA^
EBL1	43.37 ± 2.50^hijC^	49.25 ± 3.96^gB^	62.03 ± 4.26^lA^
MBL0.125	53.96 ± 2.08^bC^	67.39 ± 1.31^cB^	144.24 ± 7.79^cdA^
MBL0.25	52.11 ± 2.70^bcC^	61.77 ± 2.76^deB^	123.49 ± 7.69^fgA^
MBL0.5	47.74 ± 3.19^d–gB^	54.68 ± 5.34^fB^	85.81 ± 4.91^ijA^
MBL1	49.63 ± 2.94^c–fC^	58.10 ± 2.41^efB^	74.71 ± 5.14^kA^
EBF0.125	50.32 ± 6.76^b–eC^	66.06 ± 4.50^cdB^	147.80 ± 9.29^bcA^
EBF0.25	51.92 ± 2.48^bcdC^	67.96 ± 3.30^cB^	122.60 ± 7.16^fgA^
EBF0.5	47.52 ± 2.52^e–hB^	58.04 ± 5.44^efB^	107.40 ± 9.92^hA^
EBF1	47.31 ± 2.37^e–hC^	59.36 ± 5.30^efB^	86.82 ± 5.55^ijA^
MBF0.125	50.32 ± 1.79^b–eC^	74.86 ± 3.17^bB^	155.28 ± 8.74^bA^
MBF0.25	46.70 ± 3.36^e–iC^	67.50 ± 2.67^cB^	130.70 ± 11.57^efA^
MBF0.5	47.69 ± 2.09^efgC^	56.69 ± 3.67^fB^	83.50 ± 4.12^jkA^
MBF1	43.69 ± 2.42^g–jC^	49.09 ± 2.44^gB^	61.65 ± 4.03^lA^

*Note*: Mean ± SD; ^a–g^Within a column, values superscripted with different letters are significantly different (*p* < .05). ^A–C^Within a row, values superscripted with different letters are significantly different (*p* < .05).

While LPO values obtained in the treatments containing ethanolic extract of BL were similar to the value obtained in BHT treatment on processing day, LPO values of the treatment containing 1% ethanolic BL extract were found to be lower than the values obtained in BHT treatment on the first day of storage (*p* < .05). It was observed that LPO values obtained in the treatments containing the other incorporation ratios of ethanolic BL extract were similar to the values obtained in BHT treatment.

According to LPO results obtained at the end of storage, LPO values of EBL0.125 were higher than the values determined in BHT treatment (*p* < .05), which had similar LPO values determined in EBL0.25. On the other hand, LPO values obtained in EBL0.5 and EBL1 treatments were lower than the values of BHT treatment (*p* < .05). Furthermore, LPO values obtained in MBL0.125 were higher than the values obtained in BHT treatment on all storage days (*p* < .05). While LPO values obtained in MBL0.25 were higher than the values obtained in BHT treatment on processing day (*p* < .05), LPO values were found to be at similar levels with those obtained in BHT treatment during the storage period. In MBL0.5 and MBL1 treatments, LPO values were found to be similar to those of BHT treatment on processing day and the first day of storage, LPO values were lower than those obtained in BHT treatment at the end of storage (*p* < .05). As far as the treatments containing ethanolic extracts of BF are concerned, LPO values obtained in EBF0.125 and EBF0.25 treatments on processing day and the first day of storage were higher than the values obtained in BHT treatment (*p* < .05), whereas the values obtained in EBF0.5 and EBF1 were found to be at similar levels with the values determined in BHT treatment. At the end of storage, LPO value obtained in EBF0.125 was higher than the value obtained in BHT treatment (*p* < .05), whereas the values obtained in EBF0.25 and EBF0.5 were similar to those obtained in BHT treatment. Furthermore, lower LPO values were obtained in EBF1 compared to BHT treatment at the end of storage (*p* < .05). In the treatments containing methanolic extracts of BF, LPO value obtained only in MBF0.125 was higher than the values of BHT treatment on processing day, meantime, LPO values obtained in MBF0.125 and MBF0.25 were higher than those determined in BHT treatment on the storage period (*p* < .05). Even though similar levels of LPO were obtained in MBF0.5 and BHT treatment on both the processing day and the first day of storage, lower LPO levels were determined in MBF0.5 compared to BHT treatment at the end of the storage (*p* < .05). In general, MBF1 had lower LPO compared to BHT treatment (*p* < .05). A gradual increase in LPO values was determined in all treatments (except EPP1) during storage (*p* < .05). Similarly, Turgut et al. (2017) reported that peroxide values increased during storage in control and meatballs containing PP extract. On the other hand, no significant change was observed in LPO obtained in EPP1 during storage. Although increasing the incorporation ratio of the ethanolic PP extract up to 0.5% gradually decreased LPO (*p* < .05), no further reduction in LPO was observed between incorporation ratios of 0.5% and 1%. At the end of storage, results revealed that LPO values decreased with an increasing the extract incorporation ratio in all treatments containing BL or BF and the treatments containing the methanolic PP extract (*p* < .05). Similarly, Jahan et al. (2018) also reported a decrease in the peroxide values of the meatballs as a result of an increase in the ratio of added PP extract.

#### 3.2.4 The p‐anisidine value analysis results

The results of the changes in p‐anisidine values determined during the storage of cooked beef mince at 4°C are presented in Table 6. The highest p‐anisidine values were obtained in control during the storage period (*p* < .05). An increase in p‐anisidine values indicates decomposition of primary lipid oxidation products (hydroperoxides) into secondary oxidation products (carbonyls), suggesting an advanced stage of lipid oxidation and potential quality deterioration in the meat products (Topuz et al., 2015). On processing day, p‐anisidine values in all treatments containing ethanolic or methanolic PP extracts, EBL1, MBL1, EBF1, and MBF1 treatments were lower than p‐anisidine values of BHT treatments (*p* < .05). Meantime, p‐anisidine values determined in EBL0.125, EBL0.25, EBL0.5, MBL0.125, MBL0.25, MBL0.5, EBF0.25, EBF0.5, and MBF0.5 treatments were quite similar to BHT treatment. On the other hand, higher p‐anisidine values were determined in EBF0.125, MBF0.125, and MBF0.25 compared to BHT treatment. (*p* < .05). On the first day of storage, except MPP0.125, p‐anisidine values of all treatments containing PP extract were lower than the values determined in BHT treatment (*p* < .05), while p‐anisidine values of the treatments containing ethanolic or methanolic extracts of BL and BF with the incorporation ratios of 0.125 and 0.25% were higher than p‐anisidine values of BHT treatment (*p* < .05). There was no significant difference between p‐anisidine values of BHT treatment and the treatments containing 0.5% ethanolic extract of BL and ethanolic or methanolic extracts of BF. Higher p‐anisidine values were determined in MBL0.5 and lower p‐anisidine values were determined in EBL1, MBL1, and MBF1 treatments compared to BHT treatment (*p* < .05). There was no significant difference between EBF1 and BHT treatment in terms of p‐anisidine values. All of the treatments containing ethanolic or methanolic PP extracts had lower p‐anisidine values than BHT treatment at the end of storage, while p‐anisidine values of the treatments with 0.5 and 1% ethanolic or methanolic extracts of BL and BF were found to be lower than the values found in BHT treatment (*p* < .05). On the other hand, p‐anisidine values determined in EBL0.125, MBL0.125, EBF0.125, EBF0.25, and MBF0.125 treatments were found to be higher than the values determined in BHT treatment (*p* < .05). Topuz et al. (2015) reported that addition of PP extract was as much effective as BHT for retarding the formation of p‐anisidine in anchovy oil. Andrade et al. (2023) also stated that using active packaging with pomegranate extract on beef meat decreased p‐anisidine formation during refrigerated storage. Similar p‐anisidine values with BHT treatment were determined in EBL0.25 and MBF0.25. In the samples containing ethanolic or methanolic PP extracts, in general, p‐anisidine values decreased gradually with an increase in incorporation ratio up to 0.5% (*p* < .05). However, there was no difference between the treatments containing 0.5% and 1% PP extract in terms of p‐anisidine values. In the treatments containing BL and BF extracts, a gradual decrease in p‐anisidine values was observed with an increase in incorporation ratio (*p* < .05). When the plant materials were compared among themselves, while the lowest (*p* < .05) p‐anisidine values were generally obtained in PP extracts, no significant differences were found between BL and BF extract applications. When a comparison is made in terms of applied solvent type, no significant differences were generally found in terms of p‐anisidine values between the treatments containing the extracts obtained by using ethyl or methyl alcohol for all three plant materials. P‐anisidine values increased gradually during the storage period in all treatments except MPP1 (*p* < .05).

**TABLE 6:** Effects of plant extracts on p‐anisidine values in cooked ground beef during 7‐day storage at 4°C.

Treatments	Processing day	1 day	7 days
Control	6.35 ± 0.33^aC^	10.31 ± 0.60^aB^	25.14 ± 1.50^aA^
BHT	5.13 ± 0.39^cdC^	6.35 ± 0.52^ijkB^	16.47 ± 1.08^dA^
EPP0.125	4.16 ± 0.41^ijkC^	5.79 ± 0.41^lB^	11.22 ± 1.03^gA^
EPP0.25	4.24 ± 0.31^hijC^	4.97 ± 0.33^mB^	7.18 ± 0.28^iA^
EPP0.5	3.79 ± 0.31^klB^	4.01 ± 0.31^oB^	4.99 ± 0.30^jA^
EPP1	3.68 ± 0.26^lB^	4.16 ± 0.15^noA^	4.28 ± 0.32^jkA^
MPP0.125	4.42 ± 0.27^g–jC^	6.11 ± 0.29^jklB^	12.07 ± 0.54^fgA^
MPP0.25	4.08 ± 0.30^jklC^	4.65 ± 0.27^mnB^	6.40 ± 0.41^iA^
MPP0.5	4.26 ± 0.23^g–jA^	4.38 ± 0.23^noA^	4.44 ± 0.30^jkA^
MPP1	3.72 ± 0.30^lA^	4.09 ± 0.16^oA^	3.91 ± 0.23^kA^
EBL0.125	4.97 ± 0.22^deC^	8.52 ± 0.41^dB^	18.94 ± 0.89^cA^
EBL0.25	5.12 ± 0.30^cdC^	7.75 ± 0.35^efB^	16.28 ± 0.55^deA^
EBL0.5	4.86 ± 0.18^defC^	6.61 ± 0.37^hijB^	11.35 ± 0.62^gA^
EBL1	4.44 ± 0.43^f–jC^	5.15 ± 0.36^mB^	6.75 ± 0.43^iA^
MBL0.125	5.27 ± 0.29^cdC^	9.11 ± 0.30^bcB^	21.17 ± 1.39^bA^
MBL0.25	4.96 ± 0.14^deC^	7.28 ± 0.33^fgB^	15.45 ± 0.72^eA^
MBL0.5	5.05 ± 0.29^cdeC^	7.09 ± 0.33^ghB^	11.06 ± 0.63^gA^
MBL1	4.63 ± 0.32^e–hB^	5.04 ± 0.40^mB^	7.02 ± 0.28^iA^
EBF0.125	5.73 ± 0.43^bC^	9.17 ± 0.56^bB^	21.88 ± 0.80^bA^
EBF0.25	5.45 ± 0.37^bcC^	8.62 ± 0.53^cdB^	18.29 ± 0.72^cA^
EBF0.5	4.85 ± 0.13^defC^	6.75 ± 0.44^ghiB^	12.69 ± 0.64^fA^
EBF1	4.53 ± 0.34^f–iC^	6.14 ± 0.20^jklB^	9.36 ± 0.22^hA^
MBF0.125	5.81 ± 0.41^bC^	8.92 ± 0.38^bcdB^	21.48 ± 1.09^bA^
MBF0.25	5.75 ± 0.24^bC^	7.87 ± 0.35^eB^	16.57 ± 0.76^dA^
MBF0.5	5.14 ± 0.16^cdC^	6.07 ± 0.32^klB^	11.62 ± 0.30^gA^
MBF1	4.67 ± 0.12^efgB^	5.03 ± 0.49^mB^	8.94 ± 0.32^hA^

*Note*: Mean ± SD; ^a–g^Within a column, values superscripted with different letters are significantly different (*p* < .05). ^A–C^Within a row, values superscripted with different letters are significantly different (*p* < .05).

## 4 CONCLUSION

Current study results demonstrated that the extracts (0.5% and 1% ethanolic or methanolic PP extracts, and 1% methanolic BF extract) appear to have contributed to higher cooking loss in the ground beef which could be associated with decreased pH values observed in those treatments. In general, there were no differences among the treatments in terms of L* color values. The addition of PP extract reduced a* values in cooked ground beef, while BL extract enhanced redness. Furthermore, all extracts decreased a* values and increased b* values as their incorporation ratio increased. TBARS, LPO, and p‐anisidine results indicated that incorporation of PP extract provided lower lipid oxidation level compared to other extracts. Increasing extract incorporation ratios of all tested plants resulted in more effective lipid oxidation inhibition in cooked ground beef. It may be concluded that a minimum incorporation ratio of 0.125% for each tested plant extract is sufficient for prevention of lipid oxidation in meat processing, whereas more effective lipid oxidation inhibition compared to BHT can be achieved at a minimum incorporation ratio of 0.15% PP or 0.5% BL or 1% BF extracts.

## AUTHOR CONTRIBUTIONS

**Sevgi Şimşek:** Investigation (equal); methodology (equal); validation (equal). **Birol Kılıç:** Conceptualization (equal); data curation (equal); funding acquisition (equal); investigation (equal); methodology (equal); project administration (lead); supervision (equal); visualization (lead); writing – original draft (lead); writing – review and editing (lead).

## CONFLICT OF INTEREST STATEMENT

The authors declare that they have no conflicts of interest in this work.

## Data Availability

Data will be made available on request.
